# Supplementary material for: Enhanced Stability of Dimethyl Ether Carbonylation through Pyrazole Tartrate on Tartaric Acid-Complexed Cobalt–Iron-Modified Hydrogen-Type Mordenite
Source: Molecules. 2024 Mar 28;29(7):1510. doi: 10.3390/molecules29071510 (PMC11013630; doi:10.3390/molecules29071510)
Supplement: Supplementary file 1 [file molecules-29-01510-s001.zip › molecules-2862196-supplementary.pdf]

molecules

MDPI

Supplementary Materials

Enhanced Stability of Dimethyl Ether Carbonylation through Pyrazole Tartrate on Tartaric Acid-Complexed Cobalt–Iron-Modified Hydrogen-Type Mordenite

Guangtao Fu and Xinfa Dong *

Guangdong Provincial Key Laboratory of Green Chemical Product Technology, School of Chemistry and Chemical Engineering, South China University of Technology, Guangzhou 510640, China; ce202120124494@mail.scut.edu.cn

* Correspondence: cexfdong@scut.edu.cn

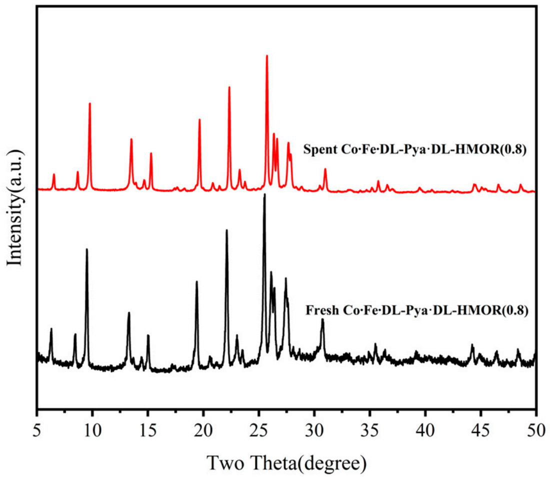

Figure S1. XRD spectrum of fresh and spent Co·Fe·DL-Pya·DL-HMOR (0.8) catalyst.

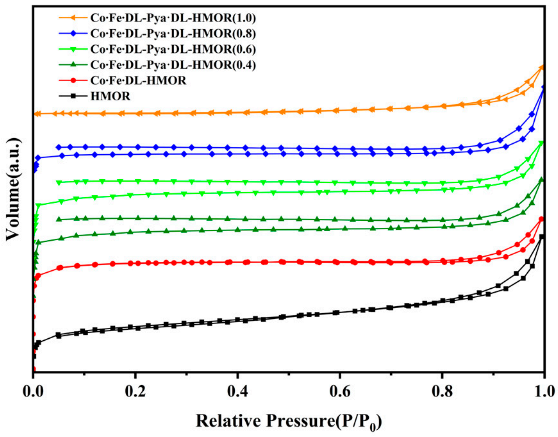

Figure S2. N_2 adsorption-desorption isotherm of HMOR, Co·Fe·DL-HMOR and Co·Fe·DL-Pya·DL-HMOR (x) (x =0.4, 0.6, 0.8, 1.0).

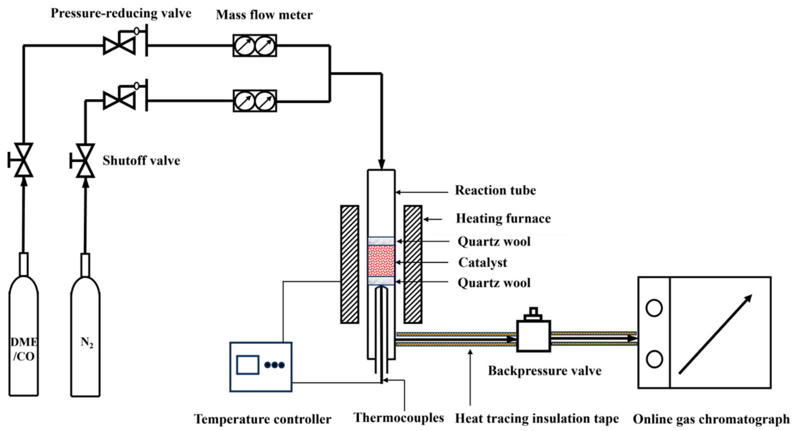

Figure S3. The schematic diagram of the catalyst testing system.

Table S1. Comparison of the DME carbonylation performance of HMOR modified by selective shielding and removal of BAS within 12-MR in recent years.

Catalysts	Conversion X_{DME}(%)	Selectivity S_{MA}(%)	Stability (h)	Reference
Co·Fe·DL-Pya·DL-HMOR (0.8)	73	>99	>400	In this work
3TMA-H-MOR	50	>99	210	[16]
1.0-[dmim]-MOR	70	>99	62	[17]
HMOR-Pya-1.3	75	>95	20	[20]
HMOR-TMCS-M	40	>99	40	[23]
Py-HMOR-6	33	>99	48	[31]
Py-MOR (Si/Al = 13.8)	85	100	20	[42]
Py-HMOR-B	36	>99	50	[43]
Py-MOR-10-673	42	>99	25	[44]
HMOR-1023	59	98	15	[45]
